# The importance of modifiable lifestyle factors for episodic memory: a gradient boosted tree analysis

**DOI:** 10.1093/geronb/gbaf225

**Published:** 2025-11-06

**Authors:** Addison Leeds Berg, Stephanie Sinclair, Ashley Acosta-Parra, César A Moreno, Ana Glosson, Rachel A Whitmer, Paola Gilsanz, Evan Fletcher

**Affiliations:** Department of Neurology, University of California, Davis, Davis, California, United States; Department of Neurology, University of California, Davis, Davis, California, United States; Department of Neurology, University of California, Davis, Davis, California, United States; Department of Neurology, University of California, Davis, Davis, California, United States; Department of Neurology, University of California, Davis, Davis, California, United States; Department of Public Health Sciences, University of California, Davis, Davis, California, United States; Division of Research, Kaiser Permanente, Oakland, California, United States; Department of Neurology, University of California, Davis, Davis, California, United States; (Psychological Sciences Section)

**Keywords:** Social determinants of health, Healthy cognitive aging, Machine learning, Exercise

## Abstract

**Objectives:**

Cognitively, physically, and socially active lifestyles are important factors of healthy aging. Our objective is to leverage novel machine learning techniques in an ethnoracially diverse population to yield powerful, explainable models of modifiable lifestyle factors contributing to memory in older adults.

**Methods:**

Cross-sectional episodic memory was the outcome in the gradient-boosted tree models and SHAP (SHapley Additive exPlanations). Participants in the Kaiser Healthy Aging and Diverse Life Experiences (KHANDLE) and the Study of Healthy Aging African Americans (STAR) made up the cohort (*n* = 2,245). Inputs included income, sex, age, education, ethnoracial groups, and 25 modifiable lifestyle factors, which were grouped into a triad of categories: leisure time activities (LTAs), physical metrics, and socialization. SHAP values indicated global feature importance and individual variability. Global importance, aggregated in each triad category, was leveraged for comparison of relative contributions to memory.

**Results:**

The global feature importance for predicting episodic memory over each of the triad categories was as follows: LTAs (0.225), physical metrics (0.169), and socialization (0.104). Each category outweighed income (0.042), ethnoracial groups (0.070), and education (0.093). LTAs had a greater importance value than sex (0.224) and age (0.203) for predicting episodic memory. Explanatory analysis depicted varying importance rankings on an individual basis.

**Discussion:**

The results confirm the importance of lifestyle variables and highlight the relative contributions of the triad categories to episodic memory in aging. This suggests that these categories may equal or surpass the importance of factors like sex, age, ethnoracial groups, and education, which are known to affect memory.

Research on healthy cognitive aging has increasingly focused on the impacts of modifiable factors that allow for early intervention (Jones et al., 2024; Ma et al., 2024; Peel et al., 2005). An influential study of these factors comes from the Lancet Commission, whose most recent report analyzes 14 modifiable factors for their contributions to dementia incidence over the life course (Livingston et al., 2024). Similarly, a comprehensive review examined the ability of seven selected factors to mitigate the effects of age and brain pathology on cognition (Song et al., 2022). Although these studies examined some of the same variables (e.g., physical activity, smoking, and alcohol consumption), they also reveal a lack of consensus on which lifestyle factors are the most relevant to healthy cognition. Moreover, the range of modifiable factors remains broad, with most studies incorporating only a subset of the variables. In the present research, we aim to refine the list of relevant modifiable factors by comparing a comprehensive set of factors available in the Kaiser Healthy Aging and Diverse Life Experiences (KHANDLE)/Study of Healthy Aging African Americans (STAR) data, encompassing many of those studied in Song and Livingston. For the purpose of our study, we define modifiable lifestyle factors to be elective lifestyle-related choices made by individuals. We acknowledge that these choices are inevitably ­constrained by structural factors beyond the individual’s control.

Past research has categorized modifiable lifestyle factors into three overarching domains—social activities, physical health and physical activities, and leisure time activities (LTAs) (Kuiper et al., 2016; Rajan et al., 2015; Wang et al., 2002). However, relatively few studies have systematically ranked the relative importance of the categories of modifiable lifestyle factors relating to cognition in diverse aging populations. Socialization, or participation in interactive events or activities, can provide insights into an individual’s mental and cognitive health (Lubben et al., 2006; Sommerlad et al., 2023). Poorer quantity and quality of social interactions have been associated with a higher risk of dementia, whereas strong social connections are associated with slowed cognitive decline (Samtani et al., 2022; Sommerlad et al., 2023). In systematic reviews and meta-analyses on social engagement, it has been found that lower levels of social isolation are associated with better later-life cognitive function and less risk of dementia (Evans et al., 2019; Penninkilampi et al., 2018). Physical activities are defined as musculoskeletal movements that expend energy above resting levels (Samtani et al., 2022). Physical health measures include sleep, grip strength, and pulmonary function (Fritz et al., 2017). Studies have shown that physical activity and health are associated with global cognition and risk of MCI and dementia (Borrell et al., 2025; Rajan et al., 2015; Stavrinou et al., 2022; Vidoni et al., 2012). Cardiorespiratory (CR) fitness has been studied as an effective method of intervention for early-stage Alzheimer’s disease, wherein declining or lower CR fitness is associated with regional brain atrophy, the progression of dementia severity, and depression symptom severity (Borrell et al., 2025; Vidoni et al., 2012). LTAs are everyday activities that are cognitively engaging, such as playing games, reading, or complex cooking (Peterson et al., 2020). Previous studies have found an association between LTA participation and cognitive function (Lee et al., 2020; Peterson et al., 2020). Cognitive reserve, the concept that individual differences in factors like educational and occupational attainment allow for better ability to manage Alzheimer’s disease pathology, has also been linked to individual differences in LTA engagement (Lee et al., 2020; Scarmeas & Stern, 2003). To build upon the findings that relate to these groups independently, we adopt a triadic framework of socialization, physical metrics (combining physical health and physical activities), and LTA categories to streamline a comparative analysis of modifiable lifestyle factor groups. Although the individual importance of each category has been investigated, it is unknown how they compare within a whole lifestyle model despite their interrelatedness.

The insights from the aforementioned studies are limited because they often focus on ethnoracially homogeneous populations and examine only one category of modifiable lifestyle factors. Additionally, previous research has commonly relied on linear analysis models, which may fail to capture more complex relationships within the data, particularly collinearity and feature interactions (Fang et al., 2025). A powerful machine learning model, incorporating the three categories of modifiable lifestyle factors together, could provide deeper insights into their importance for cognitive function in aging populations.

This paper aims to analyze the combined importance of all three categories of modifiable lifestyle factors on episodic memory function in aging individuals from diverse populations. We will employ gradient boosted tree-based machine learning models, which are structured to implicitly approximate nonlinear relationships, handle missing data, and account for feature interactions (Lundberg, Erion, & Lee, 2019). With this modeling strategy, we seek to represent cohort-wide effects from LTAs, physical metrics, and socialization. To aid in model interpretability, we will compute SHapley Additive exPlanation (SHAP) values from our model predictions to quantify these effects as global feature importance values. We aim to compare the contribution importance of these feature groups to each other and known contributors associated with episodic memory: sex, age, income, ethnoracial groups, and education. SHAP values also offer individual-level insights that highlight the variability in feature importance that population-wide investigations tend to overlook. We hypothesize that we can meaningfully rank the triad groups by global importance values, which may offer practical implications for clinical and policy-making decisions regarding cognitive aging.

## Method

### Study population

This research utilizes baseline data from the KHANDLE study and the STAR cohort, which Kaiser Permanente and UC Davis conduct. KHANDLE is a life-course study that examines the influence of early and late life factors on cognitive decline and dementia in ethnoracially diverse populations. It is composed of adults aged 65 and older as of January 1, 2017, with approximately equal representation from Black, White, Asian, and Latino(a)—hereafter Latino—self-identified ethnoracial groups, each making up roughly a quarter of the cohort. The participants are long-term Kaiser Permanente Northern California members who participated in voluntary health checkups, known as Multiphasic Health Checkups, between 1964 and 1985. All KHANDLE participants reside in the San Francisco and Sacramento regions of California.

STAR is a longitudinal study of Black American long-term Kaiser Permanente Northern California members aged 50 and older from the San Francisco Bay Area as of January 1, 2018. It examines the influence of early- and late-life factors on cognitive decline and dementia among Black American populations. STAR participants also took part in the MHC between 1964 and 1985.

Although the data in both studies were collected in longitudinal waves, this cross-sectional analysis utilizes baseline information from all participants. For KHANDLE and STAR, participants’ electronic health records were pre-screened, ensuring they did not have dementia, neuropathological diagnoses, or life-threatening conditions that would exclude them from the study at the time of enrollment. KHANDLE and STAR are approved by Kaiser Permanente Northern California and UC Davis IRBs, and all participants provided informed consent.

### Outcome: episodic memory

Our outcome variable was an episodic memory score derived from the Spanish and English Neuropsychological Assessment Scales (SENAS), which was administered verbally in either English or Spanish (by participant preference) during the first wave of both studies. This psychometric approach consists of a set of item response theory (IRT)-based tests to evaluate cognition in multiethnoracial and multilingual cohorts. The tests for episodic memory include a 15-item word-list learning test with five learning trials and a short-delay, free recall trial after distraction. The trials are ordinal items modeled with a graded-response IRT model; these are used to generate a composite score of all trials. A more comprehensive description of the methodology can be read in Mungas et al. (2004). This method is validated as an unbiased scale in a diverse, multilingual cohort like KHANDLE (Mungas et al., 2004).

### The triad of modifiable lifestyle categories: LTAs, physical metrics, and socialization

Participants underwent physical assessments and completed demographic, socioeconomic, educational, familial, and other lifestyle data surveys. We extracted responses to lifestyle survey questions representative of each category—LTAs, physical metrics, and socialization—hereafter referred to as the triad of modifiable lifestyle factor categories. The survey questions fall under our definition of modifiable lifestyle factors from the Introduction section and represent many modifiable lifestyle factors commonly studied in the literature, though meditation and diet were unavailable in the data set (Livingston et al., 2024; Song et al., 2022). A complete list of all features and cognitive measures by triad component is listed in Supplementary Material.

Participants were asked about nine common LTAs (playing games, complex cooking, writing, etc.) and rated their frequency of participation. Physical metric questions involved various modes of assessment. Continuous pulmonary function and grip strength measures were tested. Sleep hours, general health, smoking history, and light and vigorous exercise frequency were self-reported. They also reported if they ever drink alcohol. The socialization questions used various self-report measures, including perceived social standing, estimated the number of close relationships (with friends, family, and children), reported volunteer work, and described marital status. Refer to Supplementary Material for a comprehensive list of all items comprising LTAs, physical metrics, and socialization scores; precise definitions of the self-reported features are available in Supplementary Material.

In addition to the survey questions, the included sociodemographic covariates were five extensively studied factors strongly associated with cognitive aging: sex, age, ethnoracial group, income, and education (see Supplementary Material for scales). Sex was encoded as 1 for female and 0 for male. Ethnoracial groups were composed of 4 binary variables, one-hot encoded for each self-identified group mentioned in *Study population* section. Other social determinants were unavailable for these Kaiser studies.

### Data processing

Of the 2,270 participants from the combined KHANDLE-STAR cohorts, we removed 25 individuals due to missing episodic memory scores. The gradient boosted tree model we used implicitly accounts for missing predictors, so no further participant removal was necessary. The triad features were used as model inputs, along with sex, age, ethnoracial groups, education, and income. The final cohort consisted of *n* = 2,245 participants, with 30 total features after data cleaning: nine LTAs, nine physical metrics, seven socialization features, and five sociodemographic features. Table 1 provides a complete description of the final cohort.

**Table 1.** Sociodemographic and modifiable feature description of the cohort used for modeling and analysis.

Category	White (*n* = 496)	Black (*n* = 987)	Asian (*n* = 413)	Latinx (*n* = 349)	*p*-value
**Female %**	57.8629	70.2128	53.2688	58.7393	<.0001***
**Avg age (± *SD*)**	75.96 (6.70)	70.26 (8.20)	75.31 (6.13)	75.48 (5.98)	<.0001***
**Median income**	10.0000	9.0000	10.0000	8.0000	<.0001***
**Avg education years**	15.1947	14.3923	15.5864	13.1243	<.0001***
**Avg class_freq**	1.1039	1.0689	1.1478	0.9503	.1332
**Avg game_freq**	2.2607	2.1920	2.1019	1.9453	.0296*
**Avg cooking_freq**	1.6673	1.8640	1.7524	1.9182	.0230*
**Avg writing_freq**	2.8171	2.6313	2.7888	2.3446	<.0001***
**Avg artcft freq**	1.3801	1.0838	1.2214	1.2569	.0004***
**Avg cultural_eng_freq**	1.3408	1.3916	1.2360	1.3656	.0132*
**Avg art_perf_freq**	0.6524	0.8488	0.7115	0.5000	<.0001***
**Avg club_freq**	3.4919	3.0459	3.6131	3.4073	<.0001***
**Avg reading_freq**	1.1643	1.3102	1.1845	1.4307	<.0001***
**Avg pulmonary_func**	338.1132	324.6302	340.6156	324.4452	.0691
**Avg R_grip**	25.1987	26.7479	23.5556	23.0096	<.0001***
**Avg general_health**	2.4775	2.6571	2.9656	2.6492	<.0001***
**Avg L_grip**	24.1895	25.8722	22.1070	21.6102	<.0001***
**Avg vig_exc**	1.2372	1.0543	1.1520	1.0336	.0711
**Alcohol_use %**	80.2846	61.2870	66.3438	74.7748	<.0001***
**Avg sleep_hrs**	7.0629	6.1521	6.4332	6.6272	<.0001***
**History of smoking %**	52.5355	45.1120	32.2034	48.9489	<.0001***
**Avg lte_exc**	2.9185	2.6387	2.9609	2.7069	<.0001***
**Married %**	52.4194	37.1834	65.1332	53.0086	<.0001***
**Avg social_standing**	4.1540	3.5213	4.2875	4.6667	<.0001***
**Avg children_see**	1.4329	1.7119	1.6103	1.8494	<.0001***
**Volunteer %**	50.5051	51.6227	45.2555	37.8223	<.0001***
**Avg close_rel**	3.1499	5.1843	3.4801	3.7026	<.0001***
**Avg close_friend**	4.7828	4.5483	4.5661	4.0643	.5083
**Avg socializing_freq**	1.5497	1.3711	1.7524	1.6103	<.0001***
**Avg episodic memory score**	0.2101	0.1945	0.2475	−0.0413	<.0001***

*Note*. Average values and percentages indicated by ethnic group. The *p*-value column indicates if the variable for that row varies significantly by ethnic group, tested by ANOVA.

* *p* < .05.

** *p* < .01.

*** *p* < .001.

### Modeling and analysis

The modeling process involved training and tuning the hyperparameters of gradient boosted tree models, executing episodic memory predictions, and then analyzing individual and cohort-level results.

#### Model justification

In previous research, regression models have been the common statistical method to analyze the relationship between predictor variables and an outcome variable. They provide an easily interpretable representation of the strength of an association (effect size) and confidence that the association is not a statistical anomaly (statistical significance). For simpler tasks, this method is well-suited. When modeling complex relationships involving many predictor variables, which may be collinear, and may include many interactions with respect to the outcome variable, the simplicity of regression modeling can obfuscate results.

In our research, gradient boosted tree-based models allow us to examine how all three groups of modifiable lifestyle factors predict episodic memory in the same model. The use of SHAP values to analyze the magnitude of contributions from all of the features across groups facilitates novel comparisons between groups not previously possible with linear modeling. SHAP values are computed as the independent marginal contribution from each input variable, accounting for all other inputs (Lundberg, Erion, Chen, et al., 2019). These may be interpreted at the individual prediction level or averaged across the model population. Similar to beta coefficients, mean absolute SHAP values are a global model metric for a given feature. But they differ in that a mean absolute SHAP value represents a feature’s independent contribution to the predicted outcome value, versus a beta coefficient, which is an effect size.

As SHAP values are computed with consideration of all other inputs, they implicitly account for feature interactions, yielding estimates of independent feature contributions that are reliable despite collinearity. Since, within the same model, their scales are the same, we can meaningfully aggregate mean absolute SHAP values for each category by adding the components of the category together (Lundberg, Erion, Chen, et al., 2019). This method can validate previous research by showing large importance values for each group of factors while accounting for the other groups. It also goes further by ranking those group contributions against each other and key sociodemographic factors, elucidating a more precise understanding of the role of modifiable lifestyle factors on cognitive aging.

#### Model design

The structure of decision trees depends heavily on the composition of the training data. As a result, even when using the same learning algorithm and identical hyperparameters, the model’s structure may differ if the training data changes. Nested cross-validation (CV) addresses this variability by evaluating multiple training–validation splits, producing a set of hyperparameters that is more likely to generalize well to unseen data.

**Figure 1.**
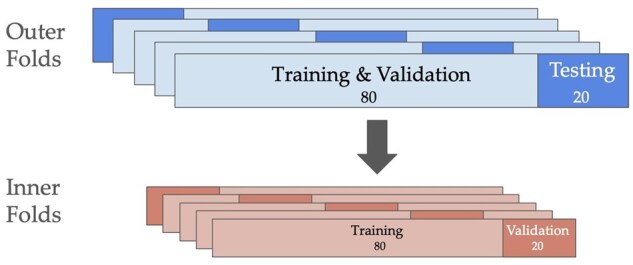
Nested 5 × 5-fold cross-validation. The diagram depicts nested 5 × 5-fold cross-validation (CV) and divides the entire cohort into five 80-20 train-test splits (outer five folds, top of image) and each of the outer folds’ training sets are divided into five 80-20 train-validate splits (inner five folds, bottom of image).

Using our data set, we applied a Bayesian hyperparameter search algorithm with nested 5 × 5-fold CV to optimize the hyperparameters of a gradient boosted decision tree model for performance and generalizability. The CV was set up to split the entire cohort into five 80-20 train-test splits (outer five folds). Each outer fold’s training set was successively split into five 80-20 train-validate splits (inner five folds). See Figure 1. The balance of the splits was defined by ensuring that the mean and variance of episodic memory scores for each split were approximately equal.

#### Hyperparameter optimization

LightGBM, an open-source gradient boosting framework that successively builds regression trees to minimize error from previous trees’ predictions, was utilized to structure our models. We used Optuna, a Bayesian algorithm framework, to efficiently search the space of model hyperparameters, optimizing our model (Akiba et al., 2019). The robust training and testing processes were performed to minimize bias from the training data, maximizing the relevance of the findings of the final model (Nielsen et al., 2024).

First, we defined a search space consisting of a broad range of model hyperparameter values so that, in trying potential combinations, our models would not risk convergence to local optima that were far from the global optimum. Hyperparameters in this space included those for learning rate, estimator (tree) count, minimum leaf samples (number of participants that defined a distinct outcome), and maximum tree depth (complexity of feature interactions considered); a complete list of hyperparameters is available in Supplementary Material. Selecting randomly from this search space, we started the first Optuna trial.

The optimization process worked by testing the current trial’s set of hyperparameters on the five distinct 80-20 train-validate splits of the five inner folds (within the current outer fold’s training set). The mean performance across all five inner folds of the current trial’s hyperparameter set was measured using an objective function (Akiba et al., 2019). In model selection, the priority of generalizability must be balanced against the priority of predictive performance. These are often in tension: hyperparameters that allow a model to closely memorize the training set may yield near-perfect accuracy but poor generalization (overfitting), whereas hyperparameters that lead to consistently poor performance across all training sets represent the opposite extreme (underfitting). Our objective function was, $\frac{(1+\mu_{v}{)}^{2}}{1+(\mu_{t}-\mu_{v}{)}^{2}}$ where $\mu_{v}$ is the mean validation *R*^2^ and $\mu_{t}$ is the mean training *R*^2^. This function balanced the maximization of predictive performance ($\mu_{v}$) and generalization (by penalizing large differences between $\mu_{t}$ and $\mu_{v}$).

Then, the next trial commenced with a new set of values for each hyperparameter, selected via Bayesian processes that considered prior trials, which is more efficient than selecting with random or exhaustive search processes (Akiba et al., 2019). Convergence among inner-fold trials yielded the optimal set of hyperparameters for each outer fold. This optimal set defined a model that was fit with the whole outer training set and performed predictions on the outer testing set (as yet, unseen data).

This process was repeated five times (for each outer fold), yielding five optimal hyperparameter sets. The final validation step was to generate 10 new 80-20 train-test splits of the entire cohort using distinct random seeds. These splits offered a way to perform a side-by-side comparison of the five optimized models on a common testbed to minimize performance variance from uniform partitioning of the data set (the previous outer splits seen in Figure 1). The model with the best average testing *R*^2^ across this final validation step was selected as the model to generate predictions for the entire cohort of SENAS episodic memory scores using the triad features and sociodemographic variables.

#### Model interpretation using SHAP values

SHapley Additive exPlanation values provide a quantitative interpretation for the weighting of inputs in the predictions (outputs) generated by tree-based models. They offer multiple levels of interpretation while illustrating variable interactions: global importance values for model inputs (analogous to Beta coefficients in regression) and individualized importances by participant (Rozemberczki et al., 2022). These are known as global and local contexts (Lundberg, Erion, Chen, et al., 2019).

Our predictions yielded SHAP values representing the marginal contribution of each input feature to the prediction of SENAS episodic memory scores, accounting for all other variables and feature interactions to the degree of complexity of the maximum tree depth (Lundberg, Erion, & Lee, 2019). This value, for a particular feature relating to a distinct prediction, is the individualized feature attribution (IFA) (Lundberg, Erion, & Lee, 2019). Global feature attributions (GFAs) were derived from the IFA magnitudes from the final optimized model, representing the average contributions for each feature across the entire cohort (mean absolute SHAP value). Calculated sums of GFAs for each category (derived from adding the GFAs for all features in a category) allowed for comparison of the triad groups with aggregate importance values. Those sums were then compared to one another and the GFAs of sex, age, income, ethnoracial groups, and education.

## Results

### Demographic characteristics

Of the 2,245 participants from the KHANDLE and STAR studies, 349 identified as Latino, 413 as Asian, 987 as Black, and 496 as White. The presence of the STAR study distinguished representation within the cohort, making the Black participant subgroup the largest ethnoracial group, with more women and a younger average age (70.26 years old). The total cohort had a higher proportion of female participants than male participants, with particularly high female representation in the Latino and Black participant subgroups (58.7% and 70.2%, respectively). The Asian participant subgroup had the highest rates of marriage (65.1%), the highest average education (15.59 years), the highest median income ($75,000 to $99,999), and the highest average SENAS episodic memory score (*z* = 0.248). In contrast, the Latino participant subgroup had the lowest average education (13.12 years), median income ($55,000 to $64,999), and the lowest average SENAS episodic memory score (*z* = −0.041). The Black participant subgroup had the lowest marriage rate (37.18%) (Table 1). All input features and episodic memory varied significantly by ethnoracial group, except for the number of close friends, pulmonary function, class frequency, and vigorous exercise frequency.

### Gradient boosted tree model selection

The model selection process was carried out as described in the *Modeling and analysis* section. The optimized model achieved an *R*^2^ of 0.392 on episodic memory predictions for the entire cohort. A full description of the hyperparameter values that defined this model is provided in Supplementary Table 1 (see online supplementary material). The gradient boosted tree model reflected improved accuracy compared to a linear regression model, including the same inputs, which achieved an adjusted *R*^2^ of 0.297.

The optimized model was used to predict episodic memory so that feature importance values could be computed. These values are shown in the beeswarm plot in Figure 3.

### Triad results

**Figure 2.**
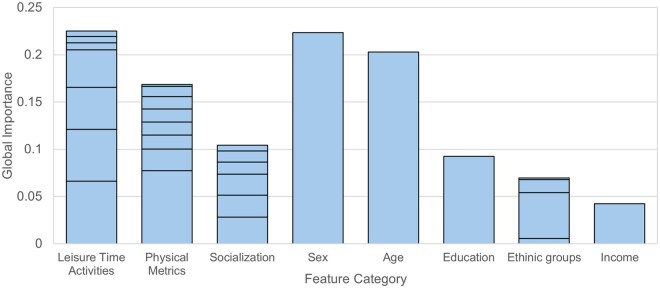
The mean absolute feature importances for each category of factor, split by individual feature. The top-down ordering of the stacked bars corresponds to the ordering of rows in Table 2. The other five bars are the mean absolute feature importances of sex, age, education, (aggregated) ethnic groups, and income.

The mean absolute feature importances (GFAs) were summed into their triad categories, yielding the following totals: LTAs 0.2253, physical metrics 0.1686, and socialization 0.1042 (Table 2). The aggregated GFAs for each category indicate the order of importance of the modifiable lifestyle factors for the cohort as (1) LTAs, (2) physical metrics, and (3) socialization. Furthermore, the importance of the top category, LTAs, was more than that of sex (0.2236) and age (0.2029). All three groups had higher GFAs than income (0.0423), ethnoracial groups (0.0695), and education (0.0925) (Figure 2).

**Table 2.** Mean absolute feature importance for each feature in the final model.

Feature	Mean absolute SHAP value
**Leisure time activities (sum)**	**0.2253**
** game_freq**	0.0663
** class_freq**	0.0547
** writing_freq**	0.0446
** cooking_freq**	0.0396
** art_perf_freq**	0.0074
** cultural_eng_freq**	0.0068
** artcft_freq**	0.0059
** club_freq**	0.0000
** reading_freq**	0.0000
**Physical metrics (sum)**	**0.1686**
** pulmonary_func**	0.0775
** general_health**	0.0228
** R_grip**	0.0147
** vig_exc**	0.0138
** alcohol_use**	0.0137
** L_grip**	0.0134
** quality_sleep_hrs**	0.0105
** smoking_history**	0.0022
** lte_exc**	0.0000
**Socialization (sum)**	**0.1042**
** social_standing**	0.0281
** children_see**	0.0233
** volunteer_work**	0.0224
** num_close_relative**	0.0125
** marital_status**	0.0120
** num_close_friend**	0.0059
** socializing_freq**	0.0000
**Sex**	**0.2236**
**Age**	**0.2029**
**Education**	**0.0925**
**Ethnic groups (aggregated)**	**0.0695**
** White ethnic group**	0.0057
** Black ethnic group**	0.0485
** Asian ethnic group**	0.0138
** Latino ethnic group**	0.0015
**Income**	**0.0423**

*Note*. SHAP = SHapley Additive exPlanations. Values in this table correspond to those represented in Figure 2. The order of the features in the table corresponds to the top-down ordering of the stacked bars for each category.

### Feature attribution results

**Figure 3.**
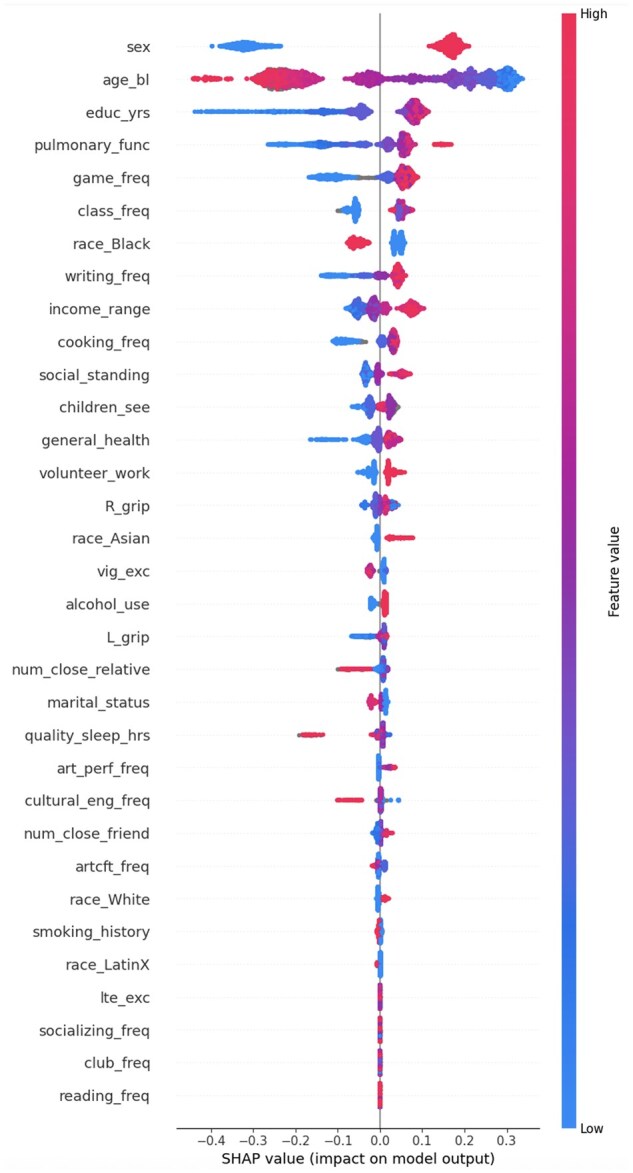
The distribution of SHAP feature importance values for each feature across the entire cohort. Feature names on the y-axis are ranked from highest to lowest mean importance from top to bottom. Dots represent the distinct Shapley additive explanation value for each participant for each feature. The feature importance is denoted on the x-axis so that a dot with a negative SHAP value falls to the left of the origin and one with a positive SHAP value falls to the right of it. The distance from the origin indicates the magnitude of feature importance, such that a dot far to the right of the origin indicates a large positive value added to the participant’s episodic memory prediction from that feature. The blue-to-red color gradient indicates the real feature value (Ex: sex = 0 is male, sex = 1 is female), grey dots indicate missing response values. As such, the continuous effect of pulmonary function is evident with low function yielding large negative SHAP values, moderate function yielding moderate positive or negative SHAP values, and high function yielding large positive SHAP values. This plot represents predictions from the model (of the 5) with the best accuracy and generalizability.

GFAs were derived from the magnitude of effect for each feature in each prediction (individual level), averaged across the entire population. The beeswarm plot (Figure 3) describes the population-level and individual-level feature importances. In this plot, the y-axis displays the relative feature importance for the whole cohort’s predictions. Thus, sex was the single most important feature, followed by age, education, and continuing to the least essential features: light exercise, socializing frequency, club frequency, and reading frequency (all mean absolute SHAP values of 0.000). The x-axis, separated at the origin, shows the SHAP values for the predictions. Each point on the plot represents an individual feature importance value (IFA) for the specified feature on the left. Points left of the origin represent negative IFAs. A negative IFA indicates that the raw value for that participant had a negative marginal contribution toward their episodic memory score prediction, thus lowering their score. Conversely, points to the right of the origin indicate positive IFAs and higher scores. The color gradient (blue to red) on the right side of the graph represents the raw feature value (low to high). Individual missing values are represented by gray points.

The effects illustrated by the beeswarm plots are best interpreted alongside the raw values for each variable, which are detailed in the Feature Scales/Measures section in Supplementary Material. For example, being female (sex = 1) positively contributed to individuals’ episodic memory scores (positive IFAs), whereas being male (sex = 0) always contributed negatively (negative IFAs). However, it is also evident that the range of SHAP values for sex among individuals is broad. Though the GFA for sex was 0.2236, the IFAs among female participants show a variable magnitude but consistently positive contribution to episodic memory; for male participants, IFAs are also variable in magnitude but consistently negative. Although the sex variable is binary, it shows a range of positive IFAs for women and a range of negative IFAs for men. Next in global importance ranking, with a GFA of 0.2029, age shows a range of IFAs from negative to positive, corresponding to the continuous raw values of this variable. Higher ages tended to have larger magnitude negative IFAs, the middle of our cohort’s age range had smaller magnitude negative or positive IFAs, and lower ages had larger magnitude positive IFAs on the prediction. The differing signs and range of magnitudes of IFAs highlight how the individuality of predictions adds nuance to the generalizations we may make about the GFAs.

## Discussion

### Overall findings

In this study of the KHANDLE and STAR cohorts, we used a LightGBM/SHAP framework to analyze modifiable lifestyle factors’ contributions to episodic memory performance at the cohort and individual levels. Previous studies have demonstrated significant linear associations between episodic memory and LTAs and similar independent associations with physical metrics and socialization on episodic memory (Liu et al., 2019; Ruthirakuhan et al., 2012; Song et al., 2022). Our global importance scores confirm these previous associations, showing that all three categories of modifiable lifestyle factors are important predictors of episodic memory in older adults.

Additionally, our framework allowed for comparison of the triad categories by feature importance, revealing the relative rankings to be (1) LTAs, (2) physical metrics, and (3) socialization (Figure 2); LTAs also surpassed single high-importance factors like sex and age. Sex was the single most critical feature, corroborating findings from previous studies (Herlitz & Yonker, 2002). All three groups exceeded the GFAs of income, ethnoracial groups, and education, which are known to be strongly associated with memory trajectories (Lyu & Burr, 2015; Stern, 2012; Wood et al., 2024). The corroboration of the global importance of each element of the triad, along with a ranking of their relative importances—both in comparison to one another and to established predictors of episodic memory—underscores the novelty of the triadic framework. The framework offers a valuable means to identify which lifestyle factor groups play the largest role in healthy cognition and may therefore serve as a guide for effective targets for cognitive health interventions.

Although the GFAs provide valuable cohort-level insights, the IFAs highlight individual variability of feature contributions to memory. Taken together, GFAs and IFAs can be useful both for clinical understanding and to guide interventions. For example, at the GFA level, the greater the variety of LTAs a person engages in, the greater the predicted impact on their cognition. These findings suggest practical applications such as the creation of community-based programs and facilities, like senior centers, supporting many forms of LTAs (Suragarn et al., 2021). Concurrently, at the IFA level, distinct details of an individual’s lifestyle could be clinically useful in developing precise interventions to support healthy cognitive aging. For example, practitioners could routinely ask patients lifestyle-related questions to determine areas of their lives where changes may benefit memory.

### Feature-level findings by category

At the cohort level, each triad category’s global importance was largely driven by a subset of key features. The degree to which these features drove their categories’ importance is detailed in the following subsections.

#### LTA feature significance

The frequency of playing games (0.0663), taking classes (0.0547), writing (0.0446), and complex cooking (0.0396) ranked among the most important individual features in the overall global feature importance ranking; the first three scores exceeded the GFA for income (0.0423). This subset of four of the nine activities within the LTA category comprised over 91.0% of the category’s GFA value (0.2253). A recent meta-analysis suggests that LTAs—including games—are positively associated with global cognition in older adults, as well as overall quality of life (Lv et al., 2025). Additional research indicates that playing games, particularly learning new complex games, may lead to changes in neuronal plasticity and strengthening (Lv et al., 2025; Maggio et al., 2018). Repeatedly participating in LTAs may strengthen cognitive reserve and recovery, memory trajectories, and may buffer the impact of age-related cognitive decline (Scarmeas & Stern, 2003).

#### Physical metrics feature significance

The physical metrics category contained a single feature (out of nine), pulmonary function (0.0775), which accounted for 46.0% of the category’s GFA (0.1686). This was the single highest GFA for a modifiable lifestyle factor. Contrasting pulmonary function’s high global feature importance, the mean absolute SHAP value for vigorous exercise was moderate (0.0138) and for light exercise it was zero. Though closely associated, the clinical precision of pulmonary function measures compared with self-reported exercise measures may have amplified the former’s contribution to predictions (Burtscher et al., 2022).

Higher baseline cardiorespiratory fitness (CR fitness; VO_2_peak) and smaller 2-year declines are associated with less AD clinical progression and less medial temporal atrophy (Vidoni et al., 2012). A systematic review of exercise trials suggests that activity can alter biomarkers, suggesting a biological basis for the CR fitness and pulmonary findings (Moniruzzaman et al., 2021). CR fitness is shaped by factors including physical activity, genetics, and smoking. Notably, prior research shows smoking lowers CR fitness and pulmonary function, reinforcing the value of counseling on smoking alongside physical activity. In inactive older adults, higher CR fitness produced patterns opposing typical aging in several brain regions, suggesting protection for memory retrieval, attention, and impulse control (Kundu et al., 2021).

#### Socialization feature significance

Three higher importance features accounted for 70.8% of the socialization category’s GFA (0.1042): social standing (0.0281), the number of their children a participant sees every month (0.0233), and self-reported volunteer work (0.0224), corroborating previous studies (Jennings et al., 2022; Kim et al., 2021; Liu et al., 2019; Zhang & Fletcher, 2021). Additionally, the number of close relatives (0.0125) and marital status (0.0120) were moderately important features, making up 23.5% of the total category’s GFA. Previous research investigating the effect of one’s social network and the relation between Alzheimer’s pathology and cognitive function indicated that social network size modified the association between the two variables (Bennett et al., 2006; Evans et al., 2019; Penninkilampi et al., 2018). For participants with larger network sizes, cognitive function remained higher despite having more severe levels of disease pathology. Our results validate the importance of the number of people in one’s social network, as evidenced in Lubben’s social scale (Lubben et al., 2006).

It has been speculated that there is a positive association between functional connectivity of anterior-posterior brain regions and social network size, and particularly, social embeddedness (Joo et al., 2017; Stam et al., 2006). Our present study corroborates the notion that social embeddedness is an important feature of socialization and may be an avenue to strengthen connectivity of functional networks that may promote healthy cognitive aging (Joo et al., 2017).

### Strengths and limitations

One limitation of our study is that the baseline data from the KHANDLE and STAR cohorts used in these analyses represent a particular aging, diverse, and cognitively normal population in Northern California belonging to the Kaiser Permanente health care system. This means that generalizing the GFAs as a reliable guide for ranking the best modifiable behaviors for healthy cognitive aging in a broader population could reflect biases from the cohort makeup. Another limitation is that much of the modifiable lifestyle factor data was self-reported, which can be a biased health data source. Conversely, the quality of our data also indicates the strength of the research. The ethnoracially diverse population improves upon studies of more homogeneous groups. Additionally, the cognitive normalcy of the cohort allows for more fine-grained measurements of episodic memory performance that could be useful for early detection and intervention.

It is important to note that the cross-sectional nature of this experiment leaves open the possibility of reverse causality, wherein poorer cognition could be a limiting factor for modifiable behavior participation. For example, there is evidence that quitting a hobby may be an early indicator of dementia (Verghese et al., 2003). However, participants were cognitively normal at the baseline for KHANDLE and STAR, which should limit the severity of this phenomenon.

Our study contains other considerable strengths. First, our modeling strategy’s ability to account for feature interaction and handle collinearity improved the robustness of feature comparisons that previous linear regression-based studies were unable to address. Second, we analyzed the triad of factors together, enabling direct comparison of their relative contributions to episodic memory. Each category maintained a large impact on episodic memory, affirming independent findings from past research on each category, together in one model (Kuiper et al., 2016; Rajan et al., 2015; Wang et al., 2002). The importance of these categories and the ability to compare them directly validates the use of our groupings and modeling strategy to represent modifiable lifestyle factors relevant to memory.

### Future work

Addressing current study limitations could broaden the scope and impact of this research. Using other large, generalizable cohorts could reinforce the findings on the relative feature importance at the population level. Longitudinal data with cognitive testing and behavioral reporting at multiple time points could track the effect of modifiable lifestyle factors on episodic memory performance over time, improving causal inference. Previously, randomized-control trials (RCTs) have supported causal effects from modifiable lifestyle factors on healthy cognitive aging. The ENGAGE protocol showed cognitive benefits in older adults from cognitively stimulating classes when paired with strategy training (Belleville et al., 2019). I-CONECT delivered semi-structured home conversations that showed evidence for cognitive function improvement (Dodge et al., 2024). Though our work cannot support causal claims, our results alongside these RCTs should encourage future work on the causal relationship between modifiable lifestyle factors and healthy cognitive aging.

The findings of the gradient-boosted tree model could be validated using other modeling strategies. Factor analysis could delineate an underlying structure of latent factors within or between the categories of modifiable lifestyle factors, which could be investigated as predictors of cognition. Other outcomes that indicate healthy cognitive aging, such as cognitive impairment diagnosis, brain tissue volumes from MRI, DTI tract measurements, blood-based biomarkers, or different measures of cognition, could further elucidate the relationship between these potentially protective factors and healthy cognitive aging. Each of these possibilities, or a combination of them, could be investigated in future work.

### Conclusion

Our study used machine learning to delineate the relative importance of an extensive array of modifiable lifestyle factors associated with episodic memory in later life. The combined factors in each triad category revealed that LTAs, physical metrics, and socialization were important contributors to episodic memory. All of these categories outweighed the contributions of education, ethnoracial groups, and income. LTAs additionally outweighed the contribution of sex and age to episodic memory. These results align with previous findings and expand upon them to provide insightful comparisons of relative feature importance. Causal inference is beyond the scope of our method. If causality can be demonstrated in future work, these findings could inform clinical and policy-driven strategies for spare time activities as effective interventions for healthy cognitive aging. Our findings suggest that LTAs, physical metrics, and socialization are all areas worthy of intervention consideration.

## Supplementary Material

gbaf225_Supplementary_Data

## Data Availability

The raw data supporting this study’s findings are available from the senior author upon request, subject to establishing a data use agreement.
