# Supplementary material for: The importance of modifiable lifestyle factors for episodic memory: a gradient boosted tree analysis
Source: J Gerontol B Psychol Sci Soc Sci. 2025 Nov 6;81(1):gbaf225. doi: 10.1093/geronb/gbaf225 (PMC12771522; doi:10.1093/geronb/gbaf225)
Supplement: gbaf225_Supplementary_Data [file gbaf225_supplementary_data.zip › JGPS suppl Berg et al.docx]

***The Journals of Gerontology, Series B: Psychological Sciences and Social Sciences* Supplementary Material: Berg et al. The Importance of Modifiable Lifestyle Factors for Episodic Memory: A Gradient Boosted Tree Analysis.**

**Feature definitions**

*LTAs*: artcft_freq – frequency of doing arts and crafts (photography, painting, sewing, woodworking, auto mechanics, etc.), cultural_eng_freq – frequency of attending cultural events (movies, live theater, concerts, museums, etc.), cooking_freq – frequency of cooking complex meals (new recipes, multiple courses, etc.), game_freq – frequency of playing games (crosswords, cards, chess, puzzles, computer games, etc.), class_freq – frequency of taking classes where you learn new skills or subjects, writing_freq – frequency of writing (letters, journal, stories, email, etc.), art_perf_freq – frequency of participation in performing arts (singing, playing an instrument, acting, etc.), reading_freq – frequency of reading (newspapers, magazines, books, internet, etc.), club_freq – frequency of attending club meetings or religious activities

*Physical Metrics*: pulmonary function, left and right-hand grip strength, average sleep hours per night, general health, frequency of light (walking, dancing, softball, bowling, etc.) and vigorous (cycling, jogging, swimming laps, tennis, etc.) exercise, alcohol use, smoking history

*Socialization*: marital status, volunteering, children_see – how many of your children do you see at least once per month, num_close_relative – how many of your relatives do you feel close to, num_close_friend – how many of your friends do you feel close to, social_standing – where does your family stand in your community, socializing_freq – frequency of socializing with friends or family

*Sociodemographics:* Sex, age, income, education, ethnic groups – What best describes your race or ethnicity

**Feature Scales/Measures**

Participants rated frequency of LTAs, socializing, and light/vigorous exercise on a scale of 1-5, with 1 being “every day,” 2 “multiple times per week,” 3 “multiple times per month,” 4 “multiple times per year,” and 5 “never.” They also rated their general health on a scale of 1-5 (1=”excellent” to 5=”poor”). Before modeling, these Likert scaled variables were rescaled so that 0 is “never” (formerly 5), 1 is “multiple times per year” (formerly 4), 2 is “multiple times per month” (formerly 3), 3 is “multiple times per week” (formerly 2), and 4 is “everyday” (formerly 1); this applies for general health as well. This was done to improve model and graph interpretability without changing the content of response groups.

For social standing, participants rated their family’s standing in the community on a scale of 1-10 (1 being the lowest and 10 the highest standing).

The modeling strategy included refused or invalid responses to scale-type questions without treating the response as part of that scale.

Participants provided integer estimates for the number of friends they feel close to, the number of relatives they feel close to (excluding children), and the number of their children they see every month. Participants were asked whether they had volunteered in the past 12 months (yes/no) and about their marital status. Marital status response options were: 1 “married,” 2 “living with a partner as if married,” 3 “separated,” 4 “divorced,” 5 “widowed,” or 6 “never married.”

Pulmonary function was measured as forced expiratory volume. Left and right-hand grip strength was measured in kilograms with a dynamometer. Alcohol use was a binary response of if they ever drink alcoholic beverages. Smoking history was a binary response of if they have ever been a smoker (smoked over 100 cigarettes at any point in their life).

Sex was recorded as 1 “male” and 2 “female”. Age was recorded at baseline to the fourth decimal; at or above 89.0000 was recorded as 89. Income was self-reported in bins representing income ranges: 1 “less than $10,000 per year”, 2 “$10,000-14,999”, 3 “$15,000-19,999”, 4 “$20,000-24,999”, 5 “$25,000-34,999”, 6 “$35,000-44,999”, 7 “$45,000-54,999”, 8 “$55,000-64,999”, 9 “$65,000-74,999”, 10 “$75,000-99,999”, 11 “$100,000-124,999”, 12 “$125,000-149,999”, 13 “$150,000 or more”. Education was operationalized as the number of completed years of schooling. Ethnic groups were derived from the survey question, which had over 20 options that were binned into the four ethnoracial groups.

**Hyperparameter definitions**

num_leaves—maximum number of leaves or distinct outcomes per tree, feature_fraction—proportion of features randomly selected per tree, bagging_fraction—proportion of participants randomly selected per tree, bagging_freq—interval for the number of trees the current bagging fraction group will be used on until a new group of random participants is bagged, learning_rate—rate at which trees are updated, max_depth—maximum depth per tree, min_child_samples—minimum number of participants needed to define a distinct tree outcome or leaf, lambda_l1—regularization for leaf scores penalizing less-predictive features, lambda_l2—regularization for overfitting penalizing noisy data, num_boost_rounds—number of trees, min_split_gain—minimum loss reduction required to perform a tree split

**Supplementary Table 1: Full feature set model**

| num_  leaves | feature_  fraction | bagging_  fraction | bagging_  freq | learning_  rate | max_  depth | min_  child_  samples | lambda_  l1 | lambda_  l2 | num_  boost_  rounds | min_  split_  gain |
| --- | --- | --- | --- | --- | --- | --- | --- | --- | --- | --- |
| 132 | 0.52412 | 0.62732 | 2 | 0.08575 | 2 | 35 | 0.60014 | 3.30475 | 159 | 0.20652 |
